# Ankle proprioception during gait in individuals with incomplete spinal cord injury

**DOI:** 10.14814/phy2.14328

**Published:** 2019-12-27

**Authors:** Charline Dambreville, Benoit Pairot de Fontenay, Andreanne K. Blanchette, Jean‐Sebastien Roy, Catherine Mercier, Laurent Bouyer

**Affiliations:** ^1^ Centre for Interdisciplinary Research in Rehabilitation And Social Integration Quebec City QC Canada; ^2^ Department of Rehabilitation Faculty of Medicine Universite Laval Quebec City QC Canada

**Keywords:** gait, proprioception, robotized orthosis, spinal cord injury

## Abstract

**Introduction:**

Proprioception is known to be affected after a spinal cord injury (SCI). However, it is currently assessed during simple tasks that do not reflect activities of daily living. To better understand how proprioception affects movement, assessing it during a functional sensorimotor task such as walking is therefore of primary importance.

Therefore, the objectives of this study were as follows: (a) measure the protocol reliability of a new robotic test in nondisabled controls; (b) evaluate the effect nonlesion‐related factors such as sex, age, pain, and gait speed on ankle proprioception; and (c) assess ankle proprioception during walking in individuals with SCI.

**Methods:**

In the current study, ankle proprioception was assessed during gait in individuals with an incomplete spinal cord injury (iSCI; *n* = 15) using an electrohydraulic robotized ankle–foot orthosis (rAFO). Ankle proprioceptive threshold was quantified as the participants’ ability to detect torque perturbations of varied amplitude applied during swing by the rAFO. In addition, test–retest reliability and the potential effect of nonlesion‐related factors (sex, age, pain, and gait speed) were evaluated in nondisabled (ND; *n* = 65) participants.

**Results:**

During gait, individuals with iSCI had a 53% poorer proprioceptive threshold than ND controls (*p* < .05). Test–retest reliability was good (ICC = 0.78), and only gait speed affected proprioceptive threshold (*p* = .018).

**Conclusion:**

This study is the first to show that ankle proprioception assessed during gait is impaired in individuals with an iSCI. The developed test can now be used to better characterize proprioception in population with other neurological conditions and has potential to maximize functional recovery during gait training in those populations.

## Graphical abstract

Ankle proprioceptive threshold assessment during gait using an electrohydraulic ankle foot orthosis is a reliable test. Gait proprioceptive threshold is affected in individuals with an incomplete spinal cord injury, in a manner that is not related to pain, gait speed, age or sex differences. Gait speed affects ankle proprioceptive threshold during gait, while age, sex and pain have no effect.

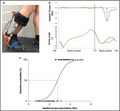

## 1 INTRODUCTION

The control of movement for the execution of activities of daily living, such as walking, requires a complex interplay between sensory and motor functions (Zwergal et al., 2012). After an incomplete spinal cord injury (iSCI), sensorimotor deficits (such as decreased sensitivity, muscle weakness) and neuropathic pain are reported (Cairns, Adkins, & Scott, 1996; Crossman, 1996), and affect the quality of life (Anneken, Hanssen‐Doose, Hirschfeld, Scheuer, & Thietje, 2010; Donnelly & Eng, 2005). While sensory and motor function deficits in individuals with an iSCI have been relatively well characterized (Curt & Dietz, 1997; Ditunno, Burns, & Marino, 2005), the effect of such lesion on the interplay between these functions, also called sensorimotor integration, is still very sparse.

Proprioception, defined as the ability to perceive body segment positions and displacements (Goble & Anguera, 2010), requires effective sensorimotor integration to aid proper movement control. It is well known that neurological pathologies such as iSCI, stroke, Parkinson disease, and neuropathy can affect proprioception (Abbruzzese, Trompetto, Mori, & Pelosin, 2014; Chisholm, Domingo, Jeyasurya, & Lam, 2016; Dietz & Fouad, 2014; Kenzie et al., 2014; Rothwell et al., 1982). As gait recovery is one of the top 3 priorities for individuals with iSCI and knowing that good proprioception is critically important to adapt the gait pattern to the environment (e.g., terrain irregularities), the current study focused on quantifying proprioceptive ability (here detection threshold) after the lesion.

As the central nervous system dynamically modulates how sensory information is processed during movement, it is not surprising that proprioception assessments performed passively or during simple single joint motion do not correlate well with motor performance during functional tasks in people with neurological impairments (Deshpande, Connelly, Culham, & Costigan, 2003; Lin, 2005). Furthermore, as motor function is influenced by sensory feedback, a characterization of proprioceptive impairment during movement (ideally in a task‐specific manner) is therefore necessary to address potential deficits and to maximize functional recovery. However, measuring proprioception during movement is not easy and cannot therefore be routinely performed in the clinic. Only passive motion detection, motion direction discrimination, and joint repositioning (actively or passively) tests are currently used in clinics to assess proprioception in individuals with iSCI (Hillier, Immink, & Thewlis, 2015).

In research laboratories, new tests have been developed by taking advantage of recent advances in robotics (Chisholm et al., 2016; Domingo, Marriott, Grave, & Lam, 2011). However, these tests are conducted during simple tasks (e.g., single joint movement) and do not capture the complexity of assessing proprioceptive capacity during dynamic movements, such as normally performed during activities of daily living. Therefore, only limited information can be provided to the clinician for the design of targeted, patient‐oriented interventions.

In the current study, an electrohydraulic robotized ankle–foot orthosis (rAFO) developed in our laboratory (Noel, Cantin, Lambert, Gosselin, & Bouyer, 2008) was used to assess ankle proprioception during gait. It has been previously validated in healthy participants (Fournier Belley et al., 2016). For the present study, a shortened version of the test was developed to optimize the evaluation of people with neurological/musculoskeletal disorders (e.g., who may suffer from pain, or present limited endurance). Its test–retest reliability will be presented as part of the results. We also investigated the contribution of factors that could influence proprioception, but that are not specific to individuals with iSCI; they are sex, age, pain, and gait speed. It is crucial to understand their impact on proprioception to determine how to take them into account in studies assessing proprioception deficits or investigating relationship between proprioception and sensorimotor functions. These factors were considered here because of the high male to female ratio (Singh, Tetreault, Kalsi‐Ryan, Nouri, & Fehlings, 2014) and, as the age of individuals with iSCI varies over a large range (Toda, Nakatani, Omae, Fukushima, & Chin, 2018), and knowing that ankle joint proprioception can be impaired in the elderly (Franco, Santos, & Rodacki, 2015; Madhavan & Shields, 2005; Skinner, Barrack, & Cook, 1984; Thelen, Brockmiller, Ashton‐Miller, Schultz, & Alexander, 1998a). Moreover, the majority of individuals with SCI experience pain (van Gorp, Kessels, Joosten, Kleef, & Patijn, 2015; Weerakkody, Blouin, Taylor, & Gandevia, 2008), and pain can affect joint position sense or movement detection threshold during static tasks (Malmstrom, Westergren, Fransson, Karlberg, & Magnusson, 2013; Weerakkody et al., 2008). Also, after an iSCI, people tend to walk at slower gait speed (van Hedel & Group ES, 2009), so it was also important to measure the effect of gait speed on proprioceptive threshold. Finally, the discriminative validity of the ankle proprioceptive threshold during gait between healthy people and individuals with iSCI was assessed and the ankle proprioception deficits were characterized in this population.

The 3 objectives of this study were to:

1. Measure the test–retest reliability of the new short duration version of the assessment tool.
2. Evaluate, in healthy participants, whether nonspecific factors to the SCI population could influence ankle proprioception results.
3. Assess ankle proprioception during gait in individuals with incomplete spinal cord injury.

## 2 MATERIAL AND METHODS

### 2.1 Participants

A total of 80 individuals participated in this study, 15 of whom had been diagnosed with an iSCI and 65 nondisabled (ND) participants. For iSCI participants, the inclusion criteria were as follows: (a) to be over 18 years of age; (b) to have an incomplete SCI (American Spinal Injury Association [ASIA] Impairment Scale (Kirshblum et al., 2011) C or D); (c) to have stable medical conditions; and (d) to be able to walk independently on a treadmill. The exclusion criteria were as follows: (a) other neurological or musculoskeletal injuries that could affect task performance.

For the ND participants, inclusion criteria were as follows: (a) to be an adult between 18 and 70 years of age; and (b) to be right foot dominant according to the Waterloo Footedness questionnaire (Elias, Bryden, & Bulman‐Fleming, 1998). The exclusion criteria were as follows: (a) self‐report of medically diagnosed chronic ankle instability; (b) musculoskeletal injury at the lower limb in the 6 months prior to the experiment; and (c) known neurological disorders or pain that could affect task performance. Of the ND participants, 25 were recruited for the assessment of test–retest reliability, and to establish the effect of pain and gait speed on proprioceptive threshold. An additional 40 participants were assessed for the effect of sex and age (see Figure 2).

All participants provided their written and informed consent prior to participating, and the study was approved by the local ethics review board.

### 2.2 Experimental protocol

**Figure 1:**
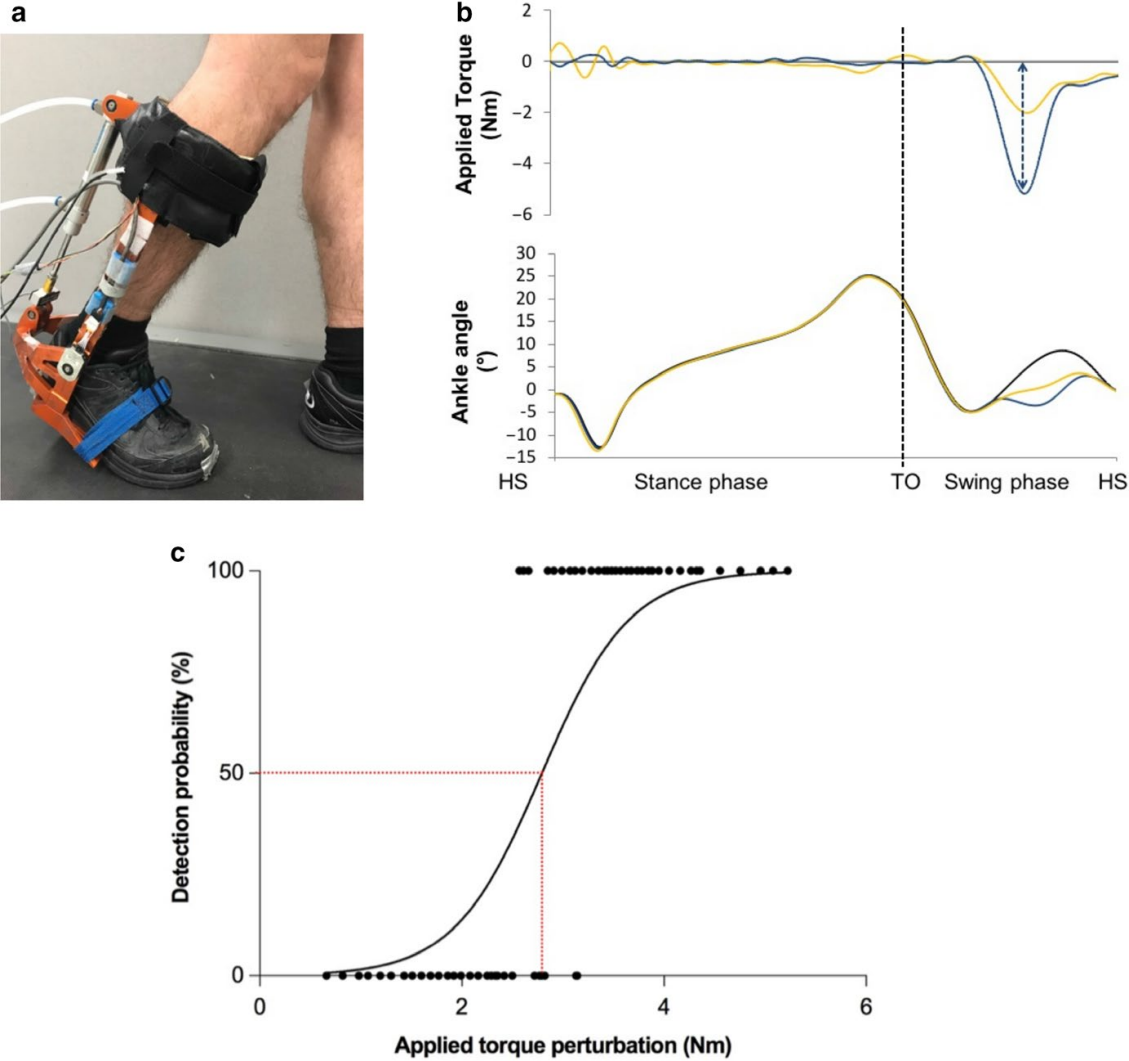
(a) Electrohydraulic robotized ankle–foot orthosis (rAFO). (b) Torque applied by the rAFO on the ankle joint (top) and ankle angle during a gait cycle (bottom; from one heel strike [HS] to the next). Black line represents the mean of the nonperturbed gait cycles; yellow and blue lines represent medium and large intensity perturbations and their kinematic consequences, respectively. The dashed blue arrow represents the maximal torque deviation caused by a large intensity perturbation. (c) Detection probability of applied torque perturbations. A sigmoidal curve (black line) was fitted to the data, and the force perturbation detection threshold was determined at the 50% detection level (dashed line)

All sessions started with the collection of participant characteristics (sex, age, weight, height). Then, participants walked on a treadmill while wearing a rAFO (see Figure 1a) on their right ankle (ND participants) or on the most affected side (according to a physiotherapist assessment) for participants with an iSCI. A familiarization period with the rAFO was given to all participants, followed by the ankle torque perturbation test (see below).

### 2.3 Ankle torque perturbation test to assess proprioceptive threshold

During gait, a torque perturbation was applied by a custom designed rAFO (Noel et al., 2008) unexpectedly at the beginning of the swing phase (60%–70% of the stride cycle), on average every 5th strides (randomized between the 3rd and 7th stride to prevent anticipation; Figure 1b). The exact timing of the torque perturbation in the gait cycle was adjusted for each participant based on their individual baseline walking pattern so that it occurs during early dorsiflexion.

The amplitude of the applied perturbation was varied to assess proprioceptive threshold, and participants were asked to press a hand‐held pushbutton whenever they perceived it.

The perturbation profile was always a bell‐shaped curve (Gaussian; (Noel et al., 2008)) to minimize synchronization of muscle spindle afferents, thereby rending the perturbations more natural‐like and not triggering stretch reflexes. Perturbation magnitude ranged from 0.5 to 8 Nm, where 0.5 Nm is the effective resolution of the device and 8 Nm is about 50% of the maximum device output capacity.

Choice of consecutive torque perturbation magnitudes was set using the Parameter Estimation by Sequential Testing (PEST) method in order to minimize the number of measurements needed to determine the ankle torque perturbation detection threshold (Taylor & Creelman, 1967). Briefly, after an initial perturbation of a set magnitude, the magnitude of the next perturbation changed depending on participants’ response: It was reduced or increased, depending if the participant detected or not detect the perturbation, respectively. This was continued until detections plateaued. For a complete description of the PEST algorithm, see Choi et al. (Choi, Jensen, Nielsen, & Bouyer, 2016).

Previous pilot data (*n* = 10) had shown that when applied during gait, the PEST method could lead to several false positives in some participants, thereby biasing estimation of their detection threshold. To reduce this effect, each torque magnitude was presented twice to three times in a row (without the participant knowing); torque amplitude was updated according to the PEST algorithm only after the same response was obtained twice (i.e., every 2 to 3 perturbations). For each proprioceptive threshold assessment, the total number of perturbations ranged from 45 to 70, thereby effectively reducing test duration compared to the original version of the test where 100 perturbations had to be applied (Fournier Belley et al., 2016).

### 2.4 Test–retest reliability

Twenty‐five ND participants (12 females, 13 males; age 22.88 ± 2.63 years; height 167.6 ± 23.6 cm; weight 67.3 ± 12.8 kg) came to the laboratory for two evaluations carried out one week apart to assess the test–retest reliability of the proprioception PEST test. The tests were performed at 3.6 km/h.

### 2.5 Effect of nonlesion‐related factors on ankle proprioception during gait

Details of the testing procedures for each factor that could influence proprioception independently of the spinal cord injury are described below.

#### 2.5.1 Effect of gait speed

The same 25 ND participants performed a series of two tests to evaluate the potential influence of gait speed. The first test was conducted at 3.6 km/h and the second at 1.8 km/h (corresponding to the average preferred speed for individuals with iSCI walking on a treadmill with an exoskeleton (Lam et al., 2015; Wirz et al., 2005)).

#### 2.5.2 Effect of pain

The same 25 ND participants also performed two additional tests to evaluate the potential effect of pain. Both were conducted at 3.6 km/h. The first was performed without pain, and the second in the presence of experimental pain induced by capsaicin cream, an experimental model of neuropathic pain (Bouffard, Bouyer, Roy, & Mercier, 2014, 2016; Mercier, Roosink, Bouffard, & Bouyer, 2017). A 1.5 cm‐wide ring of 1% capsaicin cream was applied around the right ankle (see Bouffard, Bouyer, Roy, and Mercier (2014) for more details). Pain intensity was assessed every 5 min using a numerical rating scale (where 0/10 means no pain and 10/10 means worse pain) until pain reached a plateau (generally, after 30–35 min). The second proprioception test was then performed, and participants continued to rate their pain level every 5 min during walking. The average pain level during walking for the group was 4.88 ± 2.03.

#### 2.5.3 Effect of sex and age

In order to assess the influence of sex and age, an additional 40 ND participants were recruited. For this total sample of 65 participants, 37 were females and 28 were males, with ages ranging from 18 to 70 years and average heights and weights of 167.9 ± 16.3 cm and 70.7 ± 12 kg, respectively.

### 2.6 Proprioceptive threshold in individuals with iSCI

**Figure 2:**
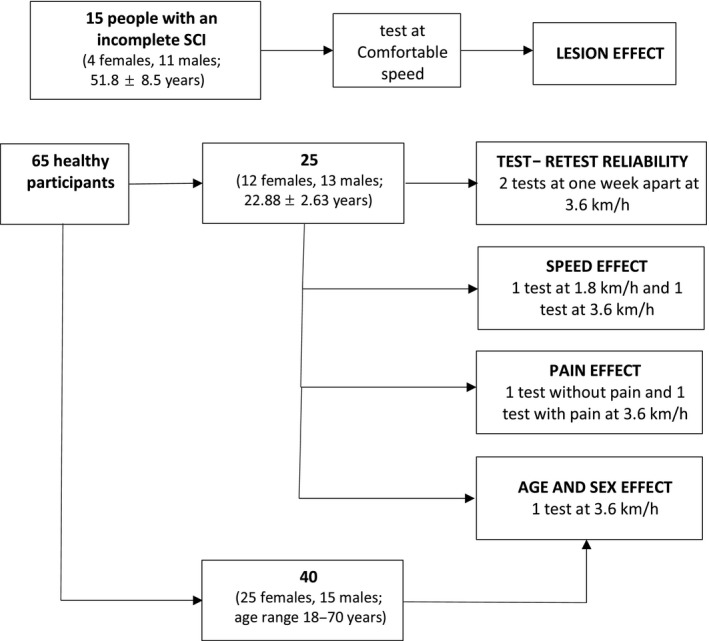
Experimental protocol for all participants

Fifteen participants with an incomplete spinal cord injury, (4 females, 11 males; 51.8 ± 8.5 years; height 176.2 ± 7.1 cm; weight 82.4 ± 17.2 kg) also participated in one evaluation of their ankle proprioceptive threshold during gait at their comfortable walking speed (Figure 2).

### 2.7 Data collection and analysis

The sagittal plane ankle angle was recorded by an optical encoder located on the rAFO, and a load cell quantified the torque applied by the rAFO on the ankle. A custom‐made foot switch recorded right heel contact (to calculate cycle duration). Pushbutton signals were also recorded.

All data were analyzed using custom‐made software written in MATLAB (The MathWorks Inc.). Using the heel contact signal, data were divided into individual gait cycles and tagged as perturbed or nonperturbed. The applied torque was calculated as the peak difference between the torque applied during the perturbation minus the residual torque present during force cancelation (mean of all nonperturbed gait cycles). For each perturbed gait cycle, applied torque and participants’ responses were extracted. A plot of response to the perturbation (100% = detected, 0% = not detected) as a function of applied torque (Nm) was then created for each participant (Figure 1c). A sigmoidal curve was fitted to the data, and the torque perturbation detection threshold (Nm) was determined as the 50% detection level (see Fournier‐Belley et al. (2016) for more details).

### 2.8 Statistics

All statistical analyses were performed using SPSS software. First, test–retest reliability for the ankle torque detection perturbation threshold was estimated by calculating intraclass correlation coefficients (ICC[3,k]) together with their 95% confidence interval (CI_95%_). ICC reliability values can be interpreted as follows: <0.20 = very poor; 0.21–0.40 = poor; 0.41–0.60 = moderate; 0.61– 0.80 = good; >0.81 = excellent (Portney, 2009). A Bland–Altman plot was created (day 1–day 2) to assess the risk of bias. The standard error of measurement (*SEM*) was calculated as follow: *SD* × √(1 – ICC), where *SD* represents the standard deviation of the measure. The minimal detectable change (MDC) was calculated as follow: z‐score (95% CI) × *SEM* × √2.

Secondly, the influence of sex, gait speed, and pain was evaluated with paired *t* tests. In addition, the strength of the correlation between participants’ age or pain score and torque perturbation detection threshold was determined using the Spearman rank correlation coefficient. Associations were classified as negligible (0.0–0.3), low (0.31–0.5), moderate (0.51–0.7), high (0.71–0.9), or very high (0.9–1.0) (Mukaka, 2012).

Thirdly, ND participants were compared to individuals with iSCI. To limit a potential oversampling bias, the group of ND participants was randomly divided in 4 groups to match the iSCI sampling size. A one‐way ANOVA was used to compare the torque perturbation detection threshold between the 5 resulting groups.

To measure the interaction between sex and age on the torque perturbation detection threshold, a two‐way ANOVA was used.

For all tests, an alpha level of 0.05 was used for statistical significance.

## 3 RESULTS

Data from 11 of the 140 tests conducted in ND participants were removed from the analyses due to technical problems that occurred during data acquisition.

### 3.1 Test–retest reliability

**Figure 3:**
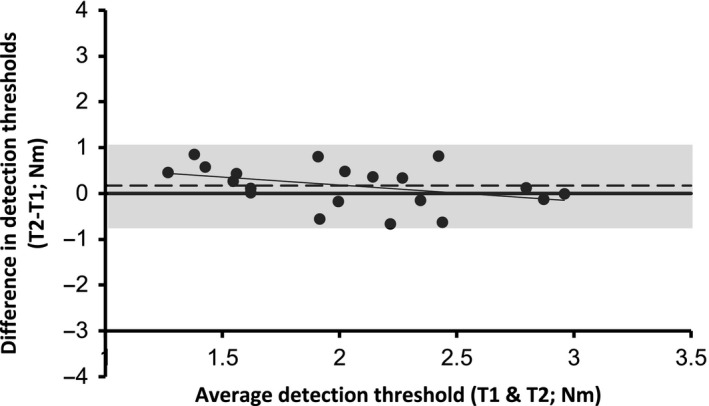
Bland–Altman plot of differences between Days 1 and 2 versus. the mean of the two measurements. The dashed line represents the mean difference, the gray line is the regression line, and the gray zone represents the mean difference ± CI_95%_

The ICC of this optimized version of the ankle torque perturbation PEST test was considered as good with a value of 0.78 (95% CI: 0.45–0.91). The *SEM* of the test was 0.38 Nm and the MDC_95%_ was 1.05 Nm. Figure 3 shows a Bland–Altman plot representing the differences between thresholds measured on Days 1 and 2 as a function of the mean of both assessments. The line of equality is located within the 95% confidence interval of the mean difference, suggesting no significant measurement bias (0.17 Nm).

### 3.2 Effect of nonlesion‐related factors on ankle proprioception

#### 3.2.1 Gait speed

**Figure 4:**
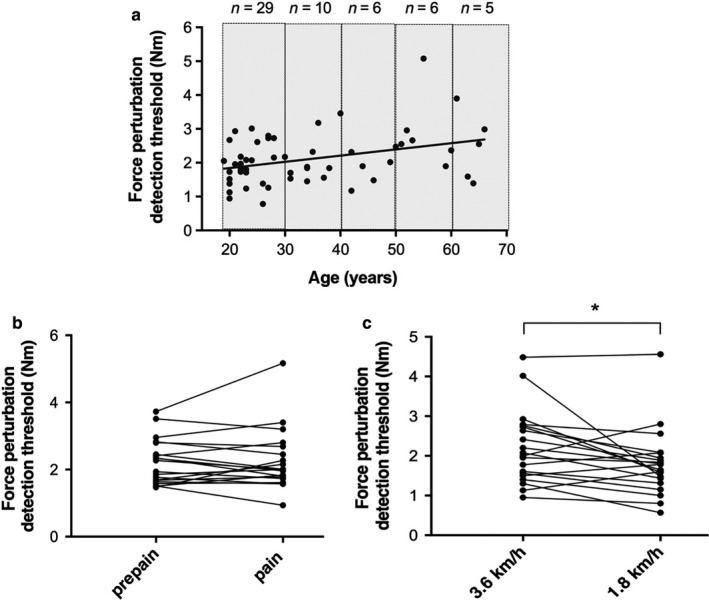
Factors that could influence proprioception. (a) Force perturbation detection threshold (Nm) as a function of age. Each dot represents the result from one participant. The n represents the number of participants in each age category (gray rectangle). The linear regression line has a Spearman rank correlation coefficient of 0.30 (*p* = .011). (b) Comparison of the force perturbation detection threshold for prepain and pain tests in all subjects (*p* = .77). Each dot represents the result from one participant. (c) Comparison of the force perturbation detection threshold between two gait speeds (3.6 km/h and 1.8 km/h) in all subjects (*p* = .018). Each dot represents the result from one participant

As shown in Figure 4c, a significant difference on the torque perturbation detection threshold was found between the two gait speeds (*p* = .018), with a lower threshold (i.e., better detection) for the slower speed (1.8 ± 0.8 Nm at 1.8 km/h and 2.2 ± 0.9 Nm at 3.6 km/h).

#### 3.2.2 Pain

As shown in the Figure 4b, no influence of pain (*p* = .77) on the torque perturbation detection threshold was found (without pain, 2.2 ± 0.7 Nm; with pain, 2.3 ± 0.9 Nm). Moreover, no correlation was found between pain score and change in torque perturbation detection threshold (r = 0.12, *p* = .3; data not shown), and no difference was measured between males and females (females, *p* = .28; males, *p* = .26).

#### 3.2.3 Sex and age

There was no influence of sex on the torque perturbation detection threshold (males: 2.1 ± 0.2 Nm; females: 2.1 ± 0.1 Nm, *p* = .53). Regarding age, while a significant (*p* = .011) statistical correlation was found between the torque perturbation detection threshold and the age of the participants, it only explained 9% of the total variance (*r* ^2^ = 0.09) (see Figure 4a). Furthermore, based on the regression equation, the estimated difference between detection thresholds at 18 and 66 years of age was 0.95 Nm, a value below the MDC_95%_ of 1.05 Nm. We therefore consider this correlation to be negligible from a clinical/functional standpoint. There was also no interaction between age and sex on the detection threshold (*p* = .23).

### 3.3 Discriminative validity in individuals with iSCI, and characterization of proprioceptive deficits

**Figure 5:**
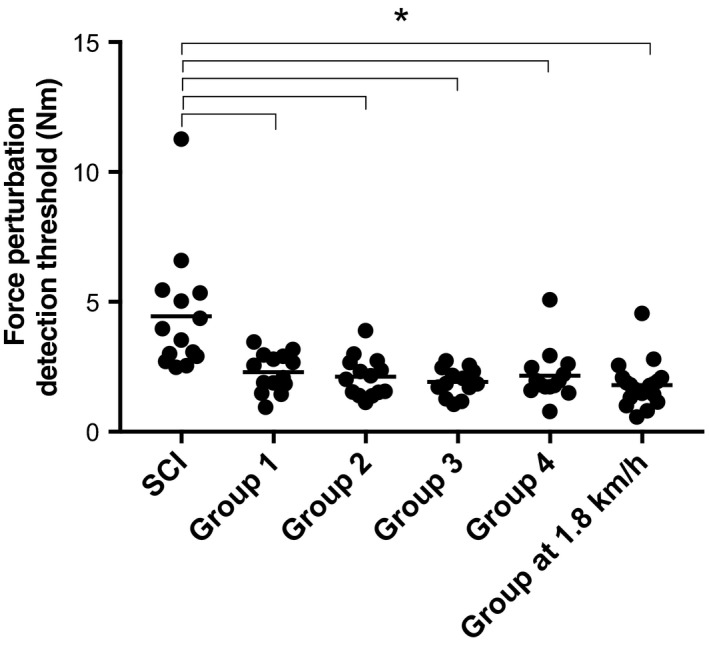
Comparison of the force perturbation detection thresholds between the SCI people and four healthy control groups (SCI vs. control groups all *p* < .003), and between SCI people and healthy group walking at 1.8 km/h (*p* < .0001). Each dot represents the force perturbation detection threshold of one participant

One participant with iSCI was excluded from the study because of his incapacity to detect any perturbation during the test, even for very large ones (>7.5 Nm). Demographic and clinical characteristics of the 14 remaining participants are presented in Table 1. To reduce oversampling bias, as the influence of age was clinically negligible (i.e., below MDC_95%_) and as there was no effect of sex, we decided to randomly divide the healthy controls (*n* = 56) into 4 groups of 14 participants (walking at 3.6 km/h) to compare with the 14 individuals with iSCI (see Statistics section above). The mean of the torque perturbation detection threshold was 4.45 ± 0.63 Nm for the iSCI group, and 2.3 ± 0.2, 2.1 ± 0.2, 1.9 ± 0.1, 2.2 ± 0.3 Nm for the 4 control groups, respectively. As shown in Figure 5, the differences between the iSCI group and the four control groups are all statistically significant (all *p* < .003). We also compared the iSCI group with the healthy subjects walking at 1.8 km/h (average walking speed of iSCI people). The difference between the 2 groups was also statistically significant (4.5 ± 0.6 Nm vs. 1.8 ± 0.2 Nm; *p* < .0001).

**Table 1:** Demographic and clinical characteristics of each people with SCI

Subject	Sex	Age (years)	Time postinjury (months)	Neurological or *anatomical* Level of injury	Comfortable speed (km/h)	Tested side	ASIA
1	F	53	Not available	T10	1.8	Right	D
2	M	54	Not available	C3‐C4	2.3	Right	D
3	M	53	5	C2	1	Right	D
4	M	45	3	L2	1.8	Right	D
5	F	35	7	C8	2.5	Left	D
6	M	48	1	L5	1.8	Right	D
7	M	58	18	L4	1.6	Right	D
8	M	48	6	C5	1.8	Left	D
9	M	60	12	C4	3	Right	D
10	M	57	9	C2	3	Left	D
11	F	53	8	T10‐T11	0.8	Right	D
12	M	44	130	C3‐C4	2.4	Left	D
13	M	71	7	C4	1.5	Right	D
14	M	47	2	T11	0.6	Right	C

## 4 DISCUSSION

The main objective of this study was to quantitatively assess ankle proprioception during gait in individuals with iSCI. To achieve this goal, control experiments were first required to assess the test–retest reliability of the protocol and the potential effect of nonlesion‐related factors on proprioception (sex, age, gait speed, and pain). Then, the discriminant validity of our test between ND participants and individuals with iSCI and the characterization of proprioceptive deficits were performed.

### 4.1 Reliability of the proprioceptive task

An optimized version of the proprioception test using the PEST algorithm was developed to reduce test duration in clinical populations. Our results show good reliability and a MDC_95%_ of 1.05Nm. The reliability was similar to that of Fournier Belley et al. (2016) with a significantly reduced test duration (now only lasting between 4 and 8 min). This optimized version of the test can now be readily used to evaluate clinical populations with potential proprioceptive deficits such as people with neurological/musculoskeletal disorders.

### 4.2 Nonlesion‐related factors that could potentially impact ankle proprioception during gait

#### 4.2.1 Sex and age

The potentially effect of sex on proprioception was measured because of the high male to female ratio for individuals with a spinal cord injury (Singh et al., 2014). No difference on the proprioceptive threshold between male and female was found in this study, thereby supporting previous work from others (Barrett, Cobb, & Bentley, 1991; Cug, Wikstrom, Golshaei, & Kirazci, 2016; Seung‐Uk, Simonsick, Deshpande, & Ferrucci, 2015).

As age of individuals with spinal cord injury varies widely (Toda et al., 2018), and proprioception was previously found to be impaired in the elderly during simple tasks (Franco et al., 2015; Kaplan, Nixon, Reitz, Rindfleish, & Tucker, 1985; Madhavan & Shields, 2005; Skinner et al., 1984; Thelen et al., 1998a), we tested the effect of age on ankle proprioceptive threshold. Our results show a significant but functionally/clinically negligible correlation between age and proprioceptive threshold. Moreover, the difference between the youngest and oldest participants, as quantified from the regression equation of Figure 4a, was below the measurement error (MDC_95%_) of 1.05 Nm. Therefore, from a clinical perspective, our results suggest that ankle proprioceptive threshold during gait is not affected by age in the range 18 to 66 years. While this finding may seem surprising, it must be remembered that ankle proprioception was assessed during gait, a complex multijoint task that is more challenging that the often‐used single joint movement in a quiet laboratory setting. Furthermore, and as discussed in depth in Fournier Belley et al. (2016), gating of sensory information is more present during complex movements than in simpler tasks. Together, these differences with previous protocols likely contribute to our results and show that functionally, ankle proprioception seems to be unaffected by age until at least 66 years old.

#### 4.2.2 Pain

Neuropathic pain (Finnerup et al., 2014) is present in the majority of individuals with SCI (van Gorp et al., 2015). It has been previously suggested that pain could alter proprioception (Malmstrom et al., 2013; Weerakkody et al., 2008). In this study, we used an experimental pain model (capsaicin cream) to simulate neuropathic pain, and no effect on proprioception during gait was reported. This might be explained by the level of pain during the task. Indeed, the mean pain level was 4.88/10 corresponding to a moderate pain whereas some studies showed that only severe pain alters proprioception (Matre, Arendt‐Neilsen, & Knardahl, 2002; Weerakkody et al., 2008). In addition, the specificity or localization of pain may have a different effect on proprioception. Indeed, proprioception is a multimodal integration of different sources of information (Proske & Gandevia, 2012). In this study, we only induced ankle cutaneous pain, leaving sensory receptors from other lower limb segments intact. They could therefore continue contributing to perturbation detection.

#### 4.2.3 Effect of gait speed

Individuals with iSCI walk at various speeds depending on their functional capacity. We therefore evaluated if walking speed, specifically slower walking speed, influences ankle proprioceptive threshold. Interestingly, we found that at slower speed, healthy subjects had a lower proprioceptive threshold, that is, had a better proprioception. It might be explained by sensory gating (see age effect above) (Saradjian, 2015), that could be more important at higher speed. The difference between the 2 speeds (0.4 Nm) remained below the MDC95% of 1.05 Nm, but this finding nevertheless suggests that at slower speeds (below 1.8 km/h), participants tend to have an even lower proprioceptive threshold. This could lead to an underestimation of proprioceptive deficits in individuals with low walking speed.

### 4.3 Assessment in individuals with iSCI

The main objective of this study was to measure ankle proprioception in individuals with an iSCI during gait using a new robotic tool. As expected, we found that ankle proprioception in individuals with iSCI is impaired compared to healthy controls, confirming and extending the scope of previous studies performed only in simple task conditions (Chang, Jung, Oh, & Kim, 2017; Chisholm et al., 2016; Domingo et al., 2011; Waters, Adkins, Yakura, & Sie, 1994). This study is the first to show the feasibility of assessing proprioception during walking in individuals with iSCI. Participants were chosen to represent a large spectrum of the demographic and clinical profiles of the individuals with an iSCI (time postinjury, walking speed, level of injury) (Chang et al., 2017). Their proprioception ranged from similar to healthy controls all the way to a lack of perturbation detection, even at the largest deviations safely possible with our device. This variability reflects the heterogeneity of the SCI population, in part due to the severity and level of injury (Burns, Marino, Flanders, & Flett, 2012). Also, we showed that gait speed during the task could influence proprioception. ND participants walked at 3.6 km/h and 1.8 km/h, and individuals with iSCI walked in the range of 0.6 to 3 km/h. Based on the reported effect of gait speed on proprioception, we may hypothesize that for the individuals with iSCI that walked at a slower speeds, the proprioceptive threshold difference with healthy control could potentially be even larger than reported here.

### 4.4 Sensory mechanism

As mentioned in the Methodology section above, the perturbation applied by the system allows a smoothly transition between the torque command send and the ankle deviation that results. This mechanical stimulation differs from perturbations produced to evoke reflex response. In the latter case, the system needs to apply rapid changes in ankle angular position and to maintain the deviated position for a short period of time. Because of its bell‐shape torque profile, the perturbation used in this study did not active only the spinal reflex pathways but has a cortical contribution too.

Sensory feedback consists of a multimodal integration of different sensory sources that have different relative weightings depending on the task and the environment (Rossignol, Dubuc, & Gossard, 2006). During walking, these sources come from muscle spindles, Golgi tendon organs, joint receptors, cutaneous mechanoreceptors and from vision or vestibular afferences (Riemann & Lephart, 2002).

Considering that the perturbation was delivered during the swing phase, sensory feedback was limited. Moreover, the perturbation was delivered at the same time for all subjects and environment. Also, gait speed and level of attention were similar across participants. Finally, participants were asked to not look at their feet during walking to avoid potential effects of visual inputs.

Measuring proprioception during walking reflects what happened during an activity of daily living and could therefore be a better test than previously used for the clinicians and patients to maximize locomotor recovery. Alternatively, it could be used in addition to static proprioceptive assessment to better characterize individual deficits.

During this experiment, the torque perturbation applied provoked an ankle angular plantar deviation. Some subjective interpretations were a pressure on the top of the foot, foot while others felt actual ankle deviations. From a mechanistic perspective, the torque perturbation likely involves cutaneous receptors from foot dorsum, muscles spindles, and Golgi tendon.

### 4.5 Strengths and limitations

The originality of this work was to assess (dynamic) ankle proprioception during a functional daily life activity (gait), whereas previous studies evaluated (static or constant joint velocity) proprioception during a single joint movement. Another contribution of this study was to evaluate the effect of potential factors that could influence ankle proprioception ability.

Some limitations need to be acknowledged however. First, some individual and/or environmental factors (e.g., physical activity, genetic factors, and lifestyle) that may affect proprioception were not considered. Second, other potential factors specific to SCI such as neuroactive drugs require further investigation. Finally, to test the applicability of this new test to the iSCI population, a wide range of lesion levels/time postinjury were selected. While this strategy showed feasibility across the iSCI population with residual gait capacity, future work on a larger sample of participant will be required to identify if specific deficits can be associated with the level of lesion.

### 4.6 Conclusion and perspectives

This study is the first to show the feasibility of assessing proprioception *during walking* in individuals with iSCI and to consider nonlesion‐related factors that could potentially influence the measure. The reliable and valid method proposed here can now be used to better characterize proprioception in individuals with iSCI under different conditions (effects of drugs, training, etc.) and also in other populations with gait deficits (Parkinson, stroke, lower limb MSK disorders, etc.).

In addition, recent studies show that (a) proprioceptive impairment affects the rate of learning a precision walking task (Chisholm, Qaiser, Williams, Eginyan, & Lam, 2019) and (b) the magnitude of improvement after gait training is related to pretraining proprioceptive sense (Qaiser, Eginyan, Chan, & Lam, 2019). The protocol developed in the present study could therefore also be used as a baseline assessment tool to potentially predict therapy outcome and help in patient screening.
